# Air Pollution and Individual and Neighborhood Socioeconomic Status: Evidence from the Multi-Ethnic Study of Atherosclerosis (MESA)

**DOI:** 10.1289/ehp.1206337

**Published:** 2013-09-27

**Authors:** Anjum Hajat, Ana V. Diez-Roux, Sara D. Adar, Amy H. Auchincloss, Gina S. Lovasi, Marie S. O’Neill, Lianne Sheppard, Joel D. Kaufman

**Affiliations:** 1 Department of Environmental and Occupational Health Sciences, University of Washington, Seattle, Washington, USA; 2 Department of Epidemiology, University of Michigan, Ann Arbor, Michigan, USA; 3 Department of Epidemiology and Biostatistics, Drexel University, Philadelphia, Pennsylvania, USA; 4 Department of Epidemiology, Columbia University, New York, New York, USA; 5 Department of Environmental Health Sciences and Risk Science Center, University of Michigan, Ann Arbor, Michigan, USA; 6 Department of Biostatistics, University of Washington, Seattle, Washington, USA

## Abstract

Background: Although research has shown that low socioeconomic status (SES) and minority communities have higher exposure to air pollution, few studies have simultaneously investigated the associations of individual and neighborhood SES with pollutants across multiple sites.

Objectives: We characterized the distribution of ambient air pollution by both individual and neighborhood SES using spatial regression methods.

Methods: The study population comprised 6,140 participants from the Multi-Ethnic Study of Atherosclerosis (MESA). Year 2000 annual average ambient PM_2.5_ and NO_x_ concentrations were calculated for each study participant’s home address at baseline examination. We investigated individual and neighborhood (2000 U.S. Census tract level) SES measures corresponding to the domains of income, wealth, education, and occupation. We used a spatial intrinsic conditional autoregressive model for multivariable analysis and examined pooled and metropolitan area–specific models.

Results: A 1-unit increase in the *z*-score for family income was associated with 0.03-μg/m^3^ lower PM_2.5_ (95% CI: –0.05, –0.01) and 0.93% lower NO_x_ (95% CI: –1.33, –0.53) after adjustment for covariates. A 1-SD–unit increase in the neighborhood’s percentage of persons with at least a high school degree was associated with 0.47-μg/m^3^ lower mean PM_2.5_ (95% CI: –0.55, –0.40) and 9.61% lower NO_x_ (95% CI: –10.85, –8.37). Metropolitan area–specific results exhibited considerable heterogeneity. For example, in New York, high-SES neighborhoods were associated with higher concentrations of pollution.

Conclusions: We found statistically significant associations of SES measures with predicted air pollutant concentrations, demonstrating the importance of accounting for neighborhood- and individual-level SES in air pollution health effects research.

Citation: Hajat A, Diez-Roux AV, Adar SD, Auchincloss AH, Lovasi GS, O’Neill MS, Sheppard L, Kaufman JD. 2013. Air pollution and individual and neighborhood socioeconomic status: evidence from the Multi-Ethnic Study of Atherosclerosis (MESA). Environ Health Perspect 121:1325–1333; http://dx.doi.org/10.1289/ehp.1206337

## Introduction

A large body of observational studies has documented associations of air pollution with health outcomes including cardiovascular disease, pregnancy outcomes, and asthma and other respiratory problems in children (Brook et al. 2010; Health Effects Institute 2010; Stieb et al. 2012). Because of the observational nature of much of the evidence, concerns are sometimes raised regarding the possibility of residual confounding by other factors. Socioeconomic status (SES) is a major concern as a cause of residual confounding because of its known links to many health outcomes (Adler and Stewart 2010) and because it is associated with air pollution exposures through class-based residential segregation and spatial clustering of air pollution sources (including traffic and point source emissions) (Gee and Payne-Sturges 2004; Mohai et al. 2009). Thus, understanding how SES and air pollution exposure are related is important when trying to infer causality based on statistical associations between air pollution and health and also has implications for a rapidly growing area of research concerning the joint effects of air pollution and SES (among other social factors) on health outcomes (Clougherty and Kubzansky 2009; Morello-Frosch and Shenassa 2006). Furthermore, characterizing the association between SES and air pollution exposures is important in order to understand the causes of disparities in many of the health conditions associated with air pollution.

Several investigations have reported associations between air pollution and SES (see Brulle and Pellow 2006). Few studies have evaluated associations between individual-level SES and air pollution (Marshall 2008). Others have examined community-level factors such as area-level poverty and educational attainment (Bell and Ebisu 2012; Brochu et al. 2011; Buzzelli and Jerrett 2007; Miranda et al. 2011; Yanosky et al. 2008). However, we are aware of only two studies that have simultaneously examined how both individual and neighborhood SES (NSES) are related to air pollution: Cesaroni et al. (2010) and Chaix et al. (2006). Both individual- and neighborhood-level SES constructs have been independently associated with health outcomes, including cardiovascular disease (Diez Roux and Mair 2010). Evidence that both constructs are independently related to air pollution would support the need to adjust for both types of measures when estimating associations between air pollution and health and would suggest that individual- and neighborhood-level socioeconomic disparities in health could be at least partly attributable to air pollution exposures.

Methodological challenges to characterizing the relation between SES and air pollution include the need to use modeling techniques that appropriately account for spatial autocorrelation (Brochu et al. 2011; Buzzelli and Jerrett 2007; Chaix et al. 2006; Jerrett et al. 2001; Yanosky et al. 2008), the need for geographic diversity (Bell and Ebisu 2012; Miranda et al. 2011), and the need for individual-level (as opposed to area-level) estimates of air pollution concentrations (Marshall 2008). Few studies of SES and air pollution have addressed these methodological issues.

A range of broader contextual factors, including the degrees of residential segregation and the spatial location of various SES groups with respect to major sources of air pollution, may modify the associations between SES and air pollution (Gee and Payne-Sturges 2004; Mohai et al. 2009). Although it is important to investigate these patterns across a range of geographic areas to properly assess heterogeneity in associations between SES and air pollution, many previous studies have been limited to a single site (Buzzelli and Jerrett 2007; Cesaroni et al. 2010; Chaix et al. 2006; Grineski et al. 2007; Jerrett et al. 2001; Marshall 2008; Molitor et al. 2011; Yanosky et al. 2008). Evidence of a lack of heterogeneity in associations between SES and air pollution would have important implications with regard to potential confounding by SES and for understanding the extent to which health disparities are attributable to air pollution.

We used data from the Multi-Ethnic Study of Atherosclerosis (MESA), a large population-based study conducted in several regions of the United States, to estimate associations of neighborhood- and individual-level SES with individual-level estimates of air pollution concentrations. We used state-of-the-art spatial modeling approaches and investigated heterogeneity across regions. MESA has collected data on an array of individual and neighborhood social factors. In addition, the study has generated individual-level space- and time-resolved estimates of airborne concentrations of particulate matter < 2.5 μm in aerodynamic diameter (PM_2.5_) and oxides of nitrogen (NO_x_). Our study hypotheses were that higher individual SES and NSES (measured by income, wealth, education, and occupation) will be associated with lower levels of individual air pollution concentrations, and that measures of NSES will be more strongly associated with individual-level air pollution concentrations than measures of individual SES.

## Methods

MESA is a longitudinal epidemiologic study designed to investigate the progression of subclinical and clinical cardiovascular disease among adults without preexisting cardiovascular disease (Bild et al. 2002). The cohort comprises 6,814 white, African-American, Hispanic, and Chinese men and women, 45–84 years of age, recruited in six communities (Baltimore, MD; Chicago, IL; Forsyth County, NC; Los Angeles, CA; New York, NY; and St. Paul, MN). The baseline examinations began in July 2000 and ended August 2002; four additional exams were conducted from 2002 until 2012. Institutional review board approval was granted at each study site, and all participants provided written informed consent. For this cross-sectional study, we included MESA participants enrolled at baseline who had complete data on PM_2.5_ or NO_x_ (the outcomes), SES characteristics (the exposures), and relevant covariates. Two MESA ancillary studies, MESA Air (Multi-Ethnic Study of Atherosclerosis and Air Polution) and the MESA Neighborhood Study, contributed data to this work.

*Air pollution.* MESA Air generated predictions of long-term ambient concentrations of PM_2.5_ (in micrograms per cubic meter) and NO_x_ (in parts per billion) for each study participant’s home address at baseline (Kaufman et al. 2012) as described elsewhere (Sampson et al. 2009; Szpiro et al. 2010). To derive these predictions, data from several sources were used: regulatory monitoring stations from the U.S. Environmental Protection Agency’s (EPA) Air Quality System (AQS), monitors deployed by MESA Air at fixed sites throughout the study area, monitors at participants’ homes, and monitors placed at specific locations to capture roadway concentration gradients (especially in the NO_x_ models) (Cohen et al. 2009). In addition, both PM_2.5_ and NO_x_ models included geographic covariates such as roadway density and land use characteristics, and outputs from dispersion models, to improve predictions. Land use covariates included population density and features such as urban land (defined as land used for residential, commercial, industrial, or transportation purposes), agricultural land, forests, and bodies of water.

The NO_x_ and PM_2.5_ estimates used in this study reflect estimated average concentrations from 1 January through 30 December 2000 at each participant’s home address at baseline. Because predicted NO_x_ values varied widely among participants (from 8.6 ppb to 173.2 ppb), we used natural log-transformed NO_x_ values as the outcome in regression models to prevent model nonconvergence. Parameter estimates for NO_x_ models were exponentiated and are presented as percentage differences from the geometric mean concentration of NO_x_. PM_2.5_ concentrations were modeled without transformation, and associations are presented as differences from the mean PM_2.5_ concentration in micrograms per cubic meter. We also performed a sensitivity analysis of associations with PM_2.5_ using annual average PM_2.5_ concentrations measured at the AQS monitor nearest to the participant’s home address at baseline as the dependent variable.

*SES*. We used SES variables from different domains (income, wealth, education, and occupation) to capture a broad conceptualization of SES, in contrast with many previous studies that have focused on only one SES domain (see Evans and Kantrowitz 2002).

Individual SES. Most individual-level SES data were collected from the baseline questionnaire. We examined income, wealth, education, and occupation as well as two additional variables (income–wealth index and working outside the home) described in the Supplemental Material, “Methods,” pp. 2–4. Total annual family income was classified based on a single question with the following 13 categorical response options: *a*) < $5,000, *b*) $5000–$7,999, *c*) $8000–$11,999, *d*) $12,000–$15,999, *e*) $16,000–$19,999, *f*) $20,000–$24,999, *g*) $25,000–$29,999, *h*) $30,000–$34,999, *i*) $35,000–$39,999, *j*) $40,000–$49,999, *k*) $50,000–$74,999, *l*) $75,000–$99,999, and *m*) > $100,000. A wealth index specified as a five-point scale (0 being the lowest level of wealth, and 4 the highest) was derived based on family ownership of four assets: homes, vehicles, land, and investments, as described previously (Hajat et al. 2010). Education was classified based on a question with the following nine categorical response options: *0*, no schooling, *1*, grades 1–8, *2*, grades 9–11, *3*, completed high school/general equivalency diploma, *4*, some college but no degree, *5*, technical school certificate, *6*, associate degree, *7*, bachelor’s degree, *8*, graduate or professional school. Occupational information was classified according to five occupational codes from the U.S. Bureau of Labor Statistics’ standard occupational categories (management/professional; service; sales/office; construction, extraction, and maintenance; and production, transportation, and material moving). Fewer than 0.2% of participants were in farming, fishing, or forestry occupations, so these participants were included in the construction, extraction, and maintenance category. Participants who were currently not working were asked to provide information on their main occupation before they stopped working. Those who had never worked outside the home and those who did not provide occupational information were excluded from the occupational classification variable. Occupation categories were collapsed into a dichotomous variable indicating management/professional occupation versus all others. The shapes of the SES–air pollution curves for family income, wealth index, and individual education were evaluated using categorical analyses (Maclure and Greenland 1992) and were found to be generally linear (data not shown). Therefore, using the 13 income categories, the 9 education categories, and the 5 wealth categories, we transformed the ordinal variables to *z*-scores, and modeled the *z*-scores as continuous variables, to facilitate comparisons with NSES variables. The *z*-scores for individual SES variables were based on the original ordinal variables, whereas the *z*-scores for NSES were based on continuous variables.

NSES. NSES metrics were obtained through the MESA Neighborhoods study. Each participant was assigned to a census tract based on their home address at baseline, and NSES-relevant domains for each census tract were characterized using 11 variables selected *a priori* from the 2000 U.S. Census (U.S. Census Bureau 2002). Income-related variables included median household income, the percentage of households living under the poverty level, the percentage receiving public assistance, and the percentage of single-parent families. Wealth-related variables included the percentage of households that own their home; the percentage that receive interest, dividend, or rental income; and the median value of owner-occupied homes. Education was characterized as the percentage of persons with at least a high school degree and the percentage with at least a Bachelor’s degree. Employment/occupation variables included the percentage unemployed and the percentage with a nonmanagerial occupation.

In addition to individual NSES variables, we used principal components analysis (PCA) with orthogonal rotation to develop a summary index to represent NSES more generally. Sixteen census variables were selected to be included in the PCA (see Supplemental Material, “Methods,” pp. 2–4, for a complete list). Factor-based scales included variables that had a factor loading of ≥ 0.6 on each factor, standardized the relevant variables, and summed them together. Based on the results of the PCA, the following six variables were included in the summary index: median household income, percentage with household income < $50,000, median value of owner-occupied homes, percentage with at least a high school degree, percentage with at least a Bachelor’s degree, and percentage with managerial/professional occupations, all of which accounts for approximately 50% of the overall variability in the original 16 variables. All NSES variables were transformed into *z*-scores. For ease of interpretation, all SES variables were scaled so that higher values indicate higher SES.

Although we evaluated a total of 11 NSES variables, we report associations of air pollution with only 6 neighborhood level characteristics here. These variables were selected to represent each of the main SES domains (wealth, income, education, and occupation). Specifically, for NSES, we selected median home value because econometric research has used home values to measure the willingness to pay for clean air (Chay and Greenstone 2005; Smith and Huang 1995). Percentage living in poverty, percentage median household income, and percentage with at least a high school degree are commonly used in the literature. Percentage with a nonmanagerial occupation potentially has implications for occupational exposure to air pollution, and, finally, the NSES index combines several important NSES variables. The variables not presented were redundant in terms of SES domain; results for these omitted variables were similar in magnitude and precision to the ones presented (data not shown).

*Covariates*. Participant age, race/ethnicity, sex, and metropolitan area were included as covariates. Models adjusted for these covariates can better inform planned epidemiological analyses because age, race/ethnicity, and sex are almost always differentially associated with health outcomes.

Models adjusted for population density and high-density land use are also presented because these variables may be associated with SES (via residential segregation) and are also predictors of air pollution. Population density was calculated as the population of the census tract divided by area of the tract not including bodies of water. Land-use data were obtained from the Multi-Resolution Land Characteristics Consortium (U.S. Geological Survey, Sioux Falls, SD) and are based on satellite data from the year 2006. We used a variable representing medium and high-density land use (defined as impervious surfaces of ≥ 50% of the total land cover) within a 100-m buffer of the participant’s address.

*Statistical analysis*. Analyses were performed using SAS, version 9.3 (SAS Institute Inc., Cary, NC), and R, version 2.14.0 (The R Foundation for Statistical Computing; Vienna, Austria). We examined the bivariate association between air pollution and SES using analysis of variance (ANOVA) and *t*-tests, and the shape of the crude air pollution–SES association was examined using LOESS curves. We used a spatial intrinsic conditional autoregressive (ICAR) model for multivariable analysis. Our model took the following form:

*y_ij_* = α + β*SES_i_* + *U_j_* + *V_ij_*, [1]

where *i* indicates the individual, *j* the census tract, *U_j_* is the spatial random effect at the census tract level, *V_ij_* is the nonspatial random effect for individuals within census tracts, and *y_ij_* is the concentration of PM_2.5_ or NO_x_ estimated at the baseline home address of individual *i* in census tract *j*. Through the *U_j_* term, the model assumes that neighboring census tracts (i.e., tracts that share a boundary) are more similar to each other than non-neighboring tracts, and that individuals within census tracts are more similar to each other than individuals living in different census tracts. To identify neighboring census tracts we created shape files (in ArcGIS; ESRI, Redlands, CA) of the census tracts where MESA participants resided, and converted the shape files into adjacency graphs in R to identify all census tracts sharing a border. Census tracts without neighbors were treated as independent in the model. ICAR models use a Bayesian framework, thus prior specifications were given for the conditional and marginal variance. Several priors were tested to ensure models were robust to changes in prior specification (see Supplemental Material, “Methods,” pp. 2–4, for more detail). Models were run with the INLA package in R (Rue et al. 2009).

Other modeling approaches were undertaken in order to compare our results to past research. Specifically, we used a multilevel model with a random intercept to account for the clustering of individuals within census tracts (see Supplemental Material, “Methods,” pp. 2–4, for more detail). Last, we conducted city-specific analyses to explore heterogeneity in associations across MESA sites.

Estimates from multivariable models adjusted only for metropolitan area (a strong confounder of the air pollution–SES association) and full models also adjusted for age, race/ethnicity, sex, metropolitan area, population density, and land use are provided here. Supplemental Material, Table S1, provides additional models, including crude models and those adjusted only for age, race/ethnicity, sex, and metropolitan area. The alpha level used to define statistical significance was set to 0.05, and we conducted complete case analysis.

## Results

MESA participants who did not consent to participate in the MESA Neighborhood Study (*n* = 623) or who reported baseline addresses outside of one of the six MESA metropolitan areas (*n* = 11) were not included in the present analysis. In addition, 40 participants did not have PM_2.5_ predictions and an additional 36 did not have NO_x_ predictions, leaving a maximum of 6,140 participants for the PM_2.5_ analysis and 6,104 participants for the NO_x_ analysis. Up to 224 participants were missing family income data, up to 350 were missing occupation data, and up to 19 were missing data for education, employment outside the home, and the wealth index.

As shown in Table 1, participants were 52% female among four race/ethnicities (39% non-Hispanic white, 27% African American, 22% Hispanic, and 12% Chinese American) with a mean age (± SD) of 61.9 ± 10.1 years and a relatively even distribution across metropolitan areas, with Los Angeles and Chicago having slightly more participants (19% and 17% respectively) than the other four sites (16%). In terms of individual SES, > 50% of participants made ≥ $40,000 per year, 55% had ≥ 3 points on the wealth index, 37% had at least a college degree, and 45% held a management occupation. The mean PM_2.5_ level across the MESA study regions was 17.2 μg/m^3^ [interquartile range (IQR), 3.4 μg/m^3^]; the mean NO_x_ level was 49.8 ppb (IQR, 40.3 ppb). As expected, for the overall study population, individuals with low SES (defined by income, wealth, education, employment, and occupation) had higher mean levels of both PM_2.5_ and NO_x_ than participants with higher SES.

**Table 1:** Mean PM_2.5_ and NO_x_ concentrations by population characteristics.

Population characteristic	Total population^*a*^ (%)	PM_2.5_			NO_x_		
		Mean (μg/m^3^)	*p*-Value^*b*^	Sample size^*c*^	Mean (ppb)	*p*-Value^*b*^	Sample size^*c*^
Sex
Female	52	17.3	0.20	6,140	50.5	0.03	6,104
Male	48	17.2			49.1
Race/ethnicity
Non-Hispanic white	39	15.8	< 0.0001	6,140	38.7	< 0.0001	6,104
Non-Hispanic black	27	17.2			49.3
Hispanic	22	18.4			65.4
Asian	12	19.9			60.1
Age (years)
45–54	29	17.0	< 0.0001	6,140	48.8	0.0005	6,104
55–64	28	17.1			48.9
65–74	29	17.4			50.5
75–84	13	17.6			52.8
Metropolitan area
Forsyth County, NC	16	16.6	< 0.0001	6,140	24.4	< 0.0001	6,104
New York, NY	16	18.3			82.6
Baltimore, MD	16	16.1			42.8
St. Paul, MN	16	12.8			26.5
Chicago, IL	17	16.3			45.1
Los Angeles, CA	19	22.3			72.4
Family income^*d*^
< $12,000	11	18.5	< 0.0001	5,916	62.0	< 0.0001	5,882
$12,000–< $25,000	19	18.4			57.9
$25,000–< $40,000	19	17.4			53.3
$40,000–< $75,000	27	16.5			44.7
≥ $75,000	24	16.5			42.5
Wealth index (points)
0 (low)	10	19.1	< 0.0001	6,139	74.2	< 0.0001	6,103
1	15	18.5			63.6
2	20	17.8			54.8
3	33	16.2			39.7
4 (high)	22	16.5			39.6
Education^*d*^
≤ High school	35	18.0	< 0.0001	6,122	57.1	< 0.0001	6,087
Some college	28	16.9			47.4
≥ College degree	37	16.7			44.9
Occupation^*e*^
Non­management	55	17.4	< 0.0001	5,790	52.7	< 0.0001	5,760
Management	45	16.8			45.4
^***a***^Denominator for percentage calculations is 6,180; this is the number of participants prior to exclusions for missing PM_2.5_ and NO_x_ data. ^***b***^*p*-Values derived from ANOVA or *t*-test. ^***c***^Sample size varies because of missing responses. ^***d***^Cate­gories of family income and education presented here have been aggregated for descriptive purposes only. ­^***e***^Participants who had never worked outside the home were excluded from the occupational classification variable (*n *= 269); participants who were currently not working were asked to provide information on their main occupation before they stopped ­working.							

**Figure 1:**
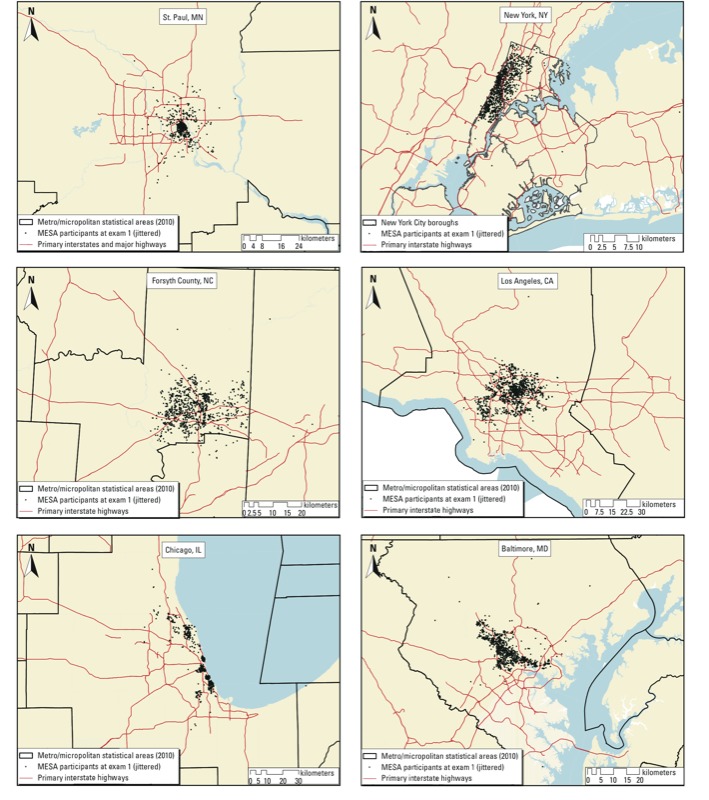
Maps of participants’ home locations at baseline in each metropolitan area. Points were randomly changed to protect participant confidentiality.

Figure 1 shows a map of each metropolitan area, marking participants’ home locations (randomly changed to protect participant confidentiality). As is evident from the maps, participants were not uniformly distributed throughout the metropolitan areas included in the study.

There were negative associations of both of the air pollutants with the individual-level SES variables in the metropolitan area–adjusted model (model 1) and the fully adjusted model (model 2) shown in Table 2, as well as the crude and minimally adjusted model shown in Supplemental Material, Table S1. Point estimates from the fully adjusted models were attenuated compared with the metropolitan area–adjusted models for the association between both pollutants and individual SES variables. For example, after adjusting for all covariates, a 1-unit increase in the *z*-score for family income (corresponding to a 1-SD unit increase in the ordinal income variable) was associated with 0.03 μg/m^3^ lower PM_2.5_ (95% CI: –0.05, –0.01) and 0.93% lower NO_x_ (95% CI: –1.33, –0.53). Associations with the wealth index and education were similar. Persons in a management occupation had 0.06-μg/m^3^ lower PM_2.5_ concentrations (95% CI: –0.09, –0.02) and 0.80% lower NO_x_ concentrations (95% CI: –1.45, –0.15) compared with persons in nonmanagement occupations.

**Table 2:** Differences from mean PM_2.5_ (95% CI) and percent difference from geometric mean of NO_x_ (95% CI) associated with an increase in individual and NSES characteristics estimated from ICAR models.^*a*^

SES variable	SD	Model 1^*b*^	Model 2^*c*^
Difference from mean PM_2.5_ (μg/m^3^) (95% CI)
Individual SES
Family income^*d*^	3.5	–0.06 (–0.07, –0.04)	–0.03 (–0.05, –0.01)
Wealth index^*d*^	1.3	–0.06 (–0.08, –0.04)	–0.03 (–0.05, –0.01)
Education^*d*^	2.4	–0.05 (–0.07, –0.03)	–0.03 (–0.05, –0.01)
Management occupation^*e*^	NA	–0.07 (–0.11, –0.04)	–0.06 (–0.09, –0.02)
NSES^*f*^
Median value of owner-occupied homes ($)	204,345	0.01 (–0.04, 0.07)	0.004 (–0.05, 0.06)
Percent not in poverty	11.4	–0.35 (–0.41, –0.28)	–0.24 (–0.3, –0.17)
Median household income ($)	20,469	–0.34 (–0.41, –0.27)	–0.25 (–0.31, –0.18)
Percent ≥ high school degree	16.7	–0.60 (–0.68, –0.53)	–0.47 (–0.55, –0.40)
Percent management occupations	17.9	–0.50 (–0.57, –0.42)	–0.38 (–0.45, –0.30)
NSES index	6.3	–0.40 (–0.47, –0.32)	–0.30 (–0.38, –0.23)
Percent difference from geometric mean NO_x_ (95% CI)
Individual SES
Family income^*d*^	3.5	–1.40 (–1.78, –1.02)	–0.93 (–1.33, –0.53)
Wealth index^*d*^	1.3	–1.58 (–1.99, –1.18)	–0.93 (–1.34, –0.53)
Education^*d*^	2.4	–1.32 (–1.69, –0.95)	–0.88 (–1.26, –0.50)
Management occupation^*e*^	NA	–1.25 (–1.92, –0.58)	–0.80 (–1.45, –0.15)
NSES^*f*^
Median value of owner occupied homes ($)	204,345	–2.86 (–3.96, –1.76)	–3.03 (–4.05, –2.02)
Percent not in poverty	11.4	–9.36 (–10.58, –8.15)	–6.72 (–7.83, –5.63)
Median household income ($)	20,469	–10.59 (–11.85, –9.34)	–7.92 (–9.04, –6.81)
Percent ≥ high school degree	16.7	–12.91 (–14.28, –11.54)	–9.61 (–10.85, –8.37)
Percent management occupations	17.9	–10.57 (–12.05, –9.10)	–7.59 (–8.91, –6.28)
NSES index	6.3	–11.39 (–12.78, –10.02)	–8.72 (–9.94, –7.50)
NA, not available. ^***a***^SES variables are scaled so that higher values indicate higher SES. ^***b***^Adjusted for metropolitan area. ^***c***^Adjusted for age, race/ethnicity, sex, metropolitan area, population density, and high-density land use. ^***d***^Parameter estimates for family income, wealth index, and education refer to a 1-unit increase in the *z*-score for these variables, which were originally ordinal variables that were transformed into *z*-scores (see Supplemental Material, “Methods,” pp. 2–4, for more details). ^***e***^Management occupation is dichotomous (management vs. non­management occupations). ^***f***^Parameter estimates for NSES variables refer to a 1-SD unit increase in that variable.			

In the population as a whole, higher NSES metrics were associated with lower concentrations of pollutants. A 1-SD increase in the percentage of persons with at least a high school degree was associated with 0.47-μg/m^3^ lower PM_2.5_ (95% CI: –0.55, –0.40) and 9.61% lower NO_x_ (95% CI: –10.85, –8.37) after covariate adjustment. Similarly, a 1-SD increase in the NSES index was associated with 0.30-μg/m^3^ lower PM_2.5_ (95% CI: –0.38, –0.23) and 8.72% lower NO_x_ (95% CI: –9.94, –7.50). In contrast, median home values were not associated with PM_2.5_ (0.004-μg/m^3^ difference from the mean with a 1-SD increase in median home values; 95% CI: –0.05, 0.06). The association between NO_x_ and median home values was substantially smaller than associations with 1-SD increases in other NSES variables, and this point estimate was not attenuated with the addition of the other covariates (–3.03% for fully adjusted model and –2.86% for metropolitan area–adjusted model, respectively). The associations between NSES characteristics and air pollutants in the crude and minimally adjusted models (see Supplemental Material, Table S1) are similar to those reported here. In general, 1-SD increases in NSES variables were more strongly associated with both PM_2.5_ and NO_x_ than corresponding increases in *z*-scores for individual-level SES factors.

Table 3 provides estimates from fully adjusted models that included both individual and NSES variables simultaneously. We used the NSES index to represent NSES, and family income, wealth index, and individual education as measures of individual SES (Spearman correlation coefficients for the NSES index and the three individual SES variables range from 0.34 to 0.43). A 1-SD increase in the NSES index was associated with 0.29-μg/m^3^ lower PM_2.5_ (95% CI: –0.37, –0.22) when controlling for family income, wealth index, and education (model D), which is essentially unchanged from the estimated association without adjustment for individual-level SES (Table 2, model 2). The individual-level SES variables were not statistically significant in model D, but still showed a negative association with PM_2.5_. NO_x_ was also negatively associated with NSES (–8.43%; 95% CI: –9.65, –7.21), family income (–0.38%; 95% CI: –0.83, 0.07), wealth index (–0.51%; 95% CI: –0.97, –0.06), and individual education (–0.47%; 95% CI: –0.87, –0.08) (model D).

**Table 3:** Differences from mean PM_2.5_ and percent difference from geometric mean of NO_x_ ­associated with a 1-SD–unit increase in SES in models including individual and NSES characteristics simultaneously.^*a*^

	NSES index^*b*^	Family income^*c*^	Wealth index^*c*^	Individual education^*c*^
SD	6.3	3.6	1.3	2.4
PM_2.5_ (μg/m^3^) (95% CI)
Model A	–0.3 (–0.37, –0.22)	–0.03 (–0.05, –0.01)	––	––
Model B	–0.3 (–0.37, –0.23)	––	–0.03 (–0.05, –0.01)	––
Model C	–0.3 (–0.37, –0.22)	––	––	–0.03 (–0.05, –0.01)
Model D	–0.29 (–0.37, –0.22)	–0.02 (–0.04, 0.01)	–0.02 (–0.04, 0.01)	–0.02 (–0.04, 0.003)
Percent change in NO_x_ (95% CI)
Model A	–8.54 (–9.77, –7.32)	–0.76 (–1.15, –0.36)	––	––
Model B	–8.59 (–9.82, –7.37)	––	–0.81 (–1.22, –0.41)	––
Model C	–8.53 (–9.76, –7.31)	––	––	–0.70 (–1.08, –0.33)
Model D	–8.43 (–9.65, –7.21)	–0.38 (–0.83, 0.07)	–0.51 (–0.97, –0.06)	–0.47 (–0.87, –0.08)
—, Variable not included in the model. ^***a***^SES variables are scaled so that higher values indicate higher SES; models adjusted for age, race/ethnicity, sex, metro­politan area, population density, and high-density land use. ^***b***^Parameter estimates for NSES index refer to a 1-SD unit increase in that variable. ^***c***^Parameter estimates for family income, wealth index, and education refer to a 1-unit increase in the *z*-score for these variables, which were originally ordinal variables that were transformed into *z*-scores (see “Methods” for more details).				

Results from multilevel models for PM_2.5_ and NO_x_ were very similar to those for the ICAR models; for several variables, both the parameter estimates and 95% CIs were almost identical (see Supplemental Material, Table S2).

**Figure 2:**
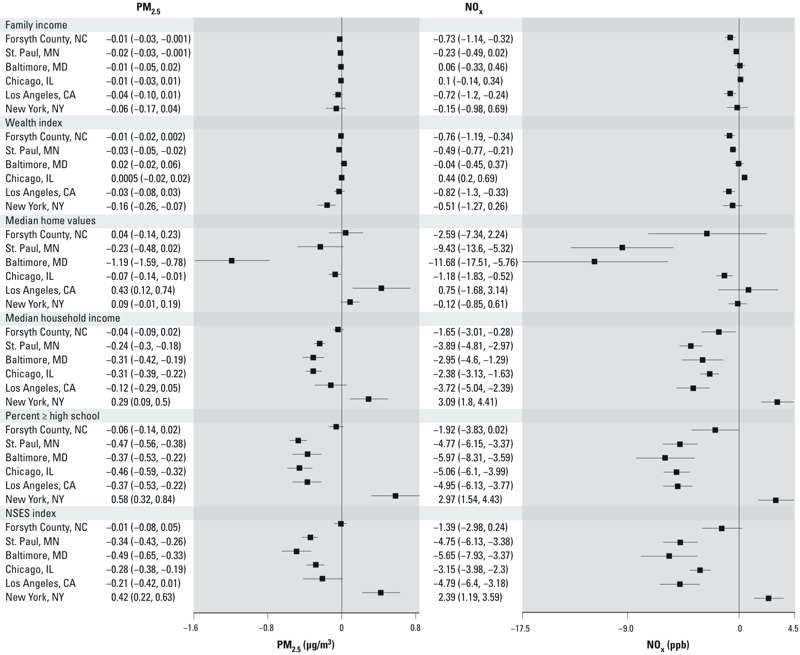
Mean differences in PM_2.5_ (μg/m^3^) and NO_x_ (ppb) concentrations (95% CI) associated with a 1-SD unit increase in SES by metropolitan area. SES variables are scaled so that higher values indicate higher SES. Models adjusted for age, race/ethnicity, sex, metropolitan area, population density, and high-density land use. Parameter estimates for family income and wealth index refer to a 1-unit increase in the z-score for these variables, which were originally ordinal variables that were transformed into z-scores (see “Methods” for more details). Parameter estimates for NSES index refer to a 1-SD unit increase in that variable.

*Metropolitan area–specific results*. Figure 2 shows considerable heterogeneity across metropolitan areas. Individual-level SES measures (family income, wealth index) were not associated with either pollutant in some cities, although negative associations consistent with the results for the population as a whole were estimated for most areas. In Chicago, however, a 1-unit increase in the individual wealth index *z*-score was positively associated with NO_x_ (0.44 ppb; 95% CI: 0.20, 0.69), indicating higher average exposures among those with higher individual SES. The association between NO_x_ and both family income and employment outside the home in Chicago were also positive, but not statistically significant (0.10 ppb; 95% CI: –0.14, 034 and 0.84 ppb; 95% CI: –0.16, 1.84 respectively). This pattern did not, however, hold for individual education and occupation (results not shown) or for PM_2.5_ in Chicago. As expected, crude models and those adjusted for age, race/ethnicity, and sex (see Supplemental Material, Table S3) showed a similar but slightly stronger association compared with the fully adjusted models shown in Figure 2.

After controlling for covariates, most cities showed negative associations between the pollutants and NSES, with the exception of New York, where both pollutants were positively associated with NSES (Figure 2). For example, a 1-SD increase in median household income was associated with 3.09-ppb higher NO_x_ (95% CI: 1.80, 4.41) and 0.29-μg/m^3^ higher PM_2.5_ (95% CI: 0.09, 0.50). Consistent with estimates for the population as a whole, city-specific differences in PM_2.5_ and NO_x_ were greater in association with 1-SD increases in NSES variables than with 1-SD increases in the individual-level measures of SES. The *z*-scores for SES variables are based on means and SDs for the overall study and are not metropolitan area specific.

## Discussion

We examined cross-sectional associations of individual and NSES with individual-level estimates of air pollutant concentrations at each participant’s residence at baseline. NSES was more strongly associated with air pollutant concentrations than were individual SES. In the overall study population, average PM_2.5_ and NO_x_ concentrations were lower as NSES increased. However, this was not the case in all city-specific analyses: We observed higher air pollutant concentrations in high-SES neighborhoods in New York.

Not only do these findings highlight the fact that associations of SES with environmental exposures may be context specific, but they also have implications for health analyses. We recommend that researchers interested in estimating associations of pollutants with health, adjusted for SES, should examine the specific associations present in their geographic regions and specific population samples before deciding how best to account for SES in their analyses.

Our findings are consistent with the previous studies of SES and air pollution (see Brulle and Pellow 2006). One-SD increases in the SES measures evaluated were associated with ≤ 0.47-μg/m^3^ lower PM_2.5_ and ≤ 4.20-ppb lower NO_x_ concentrations. These estimates represent at most approximately 4% of the annual National Ambient Air Quality Standards (NAAQS) for PM_2.5_ (12 μg/m^3^) and approximately 8% of the annual NAAQS for NO_2_ (53 ppb). Given the differences across studies, it is difficult to make direct comparisons with the literature; however, one study with a comparable range in NSES found similarly sized associations for PM_2.5_ (Brochu et al. 2011). Although associations with NSES were strong, associations between individual SES measures and air pollution were small. In models including both individual and NSES variables, the parameter estimates for 1-SD increases in the individual SES variables were several orders of magnitude smaller than corresponding associations with 1-SD increases in NSES variables. Our results are consistent with the two previous studies that have evaluated this question, finding NSES characteristics are more strongly associated with air pollutants than individual-level SES (Cesaroni et al. 2010; Chaix et al. 2006). Regardless, some caution should be exercised in interpreting these results because individual- and neighborhood-level variables were collected in different ways and from different sources.

Our data suggest that NO_x_ has a stronger association with SES than PM_2.5_. NO_x_ levels are highly dependent on proximity to busy roadways, which may also coincide with low-SES communities. In addition, the within-city variability of NO_x_ was much greater than that of PM_2.5_, further contributing to the larger magnitudes of effect sizes seen for NO_x_. Associations were measured on different scales for the two pollutants (untransformed PM_2.5_ and log-transformed NO_x_); therefore, comparisons should be made with caution.

Considerable differences in levels of air pollution and neighborhood characteristics across the six MESA metropolitan areas suggest the need to evaluate both confounding and effect modification by city. In models adjusted for metropolitan area, we found that estimated associations between PM_2.5_ and SES measures were larger than estimates from unadjusted models. This indicates that metropolitan area may negatively confound the SES–air pollution association in the overall study population. This furthermore suggests that, in epidemiologic analyses to assess the health effects of air pollution, SES may be more important for within-city contrasts than between-city contrasts.

Considerable differences in the associations between SES and air pollution were also observed in city-specific models. Lower SES was associated with higher pollution in most metropolitan areas; however, in New York, NSES measures were positively associated with PM_2.5_ and NO_x_ concentrations, suggesting higher exposures with higher SES. To our knowledge, no studies of other American cities, counties, or states have observed the pattern seen in New York, although some studies from Europe and Canada did have similar findings (Buzzelli and Jerrett 2007; Cesaroni et al. 2010). We believe these results are not caused by misclassification of air pollutant concentrations because our predictions are in line with those produced by an extensive air monitoring campaign undertaken by the New York City Department of Health and Mental Hygiene (2012). Instead, the lower-SES neighborhoods in New York included in our analyses may be somewhat isolated from roads and high-density land uses compared with high-SES areas, resulting in lower levels of traffic-related air pollution. Alternatively, high-SES neighborhoods (e.g., Upper West Side) may have higher than normal concentrations of pollutants resulting from high-density land use and proximity to busy roadways.

The definition and operationalization of a “neighborhood” is dependent on both the outcome under study and the mechanism by which the neighborhood effect is thought to operate (Diez Roux 2001; Lovasi et al. 2008). In the case of air pollution, geographically defined areas may be more relevant than social, historical, or administrative boundaries given the spatial nature of pollutants. However, our decision to use census tracts to define neighborhoods was, in part, a practical one: Confidentiality is less of a concern, more data are available at the tract level, and tracts result in fewer “islands” in the ICAR model. Furthermore, keeping in mind our objective of informing health effects analyses, we evaluated NSES metrics based on census tract data that would be readily available to other researchers. There is little evidence that block groups or spatially defined areas (e.g., 1-km buffer) better capture the true concept of a neighborhood (Lovasi et al. 2008). In our data, NSES at the census tract level is highly correlated with NSES at the block group level (correlation coefficients are ≥ 0.72 for all NSES variables). However, one limitation of using census tracts is that they vary considerably in size across metropolitan areas. For example, Winston-Salem, North Carolina, has larger and potentially more heterogeneous census tracts than other MESA sites, which suggests that census tracts may be a less meaningful way to define neighborhoods in this metropolitan area than at other study sites.

Many studies on the impact of place on health use standard multilevel models, similar to much past research on SES and environmental exposures, which relied on the entirely aspatial ordinary least squares approach (Bowen 2002). One of the present study’s strengths is that spatial regression approaches were employed and compared with aspatial multilevel models (see Supplemental Material, Table S2). In our specific application, aspatial models seem to perform as well as their more complex spatial counterparts; however, other studies have not found this to be true (Chaix et al. 2005a, 2005b; Takagi et al. 2012). Aspatial multilevel models do not consider the correlation of outcomes between areas, only within areas (Auchincloss et al. 2012). This unaccounted spatial autocorrelation may result in incorrect inference (Chaix et al. 2005a, 2005b). Our use of the spatial ICAR model overcomes this problem.

One methodological concern related to the use of ICAR models was that some census tracts that contained MESA participants had no neighbors, effectively creating “islands.” Although the ICAR models can handle this anomaly, more robust estimation would be possible with more contiguous spatial distribution of participants. In addition, recent work suggests that the way the ICAR model allows for spatial random effects may bias the fixed effects (Hodges and Reich 2010); however, this is not a concern in our study because the results from the ICAR and multilevel models were similar. One issue that statistical models cannot overcome is the spatial misalignment between the individual-level air pollution outcome and the neighborhood-level SES indicators. This has the potential to affect inference in our study. For example, the effects of living in disadvantaged neighborhoods may not increase concentrations of pollutants to which study participants are exposed as much as our NSES parameter estimates suggest. However, both the NSES variables and the pollutants are defined at and represent each participant’s residential location, which should help reduce the impact of spatial misalignment.

The associations presented in this study are not between SES and directly measured PM_2.5_ or NO_x_ but instead represent associations between SES and estimated air pollution concentrations that are predicted in part based on the covariates used to predict PM_2.5_ and NO_x_. Much of the recent research on SES and air pollution uses predicted levels of air pollution to assess the association with social factors (Brochu et al. 2011; Buzzelli and Jerrett 2007; Chaix et al. 2006; Marshall 2008; Molitor et al. 2011; Yanosky et al. 2008). Such prediction models have several benefits (e.g., leveraging multiple existing data sources), which produce more accurate predictions, thus reducing measurement error. In addition, we believe the predicted values are accurate estimates of PM_2.5_ and NO_x_ concentrations. To corroborate these results, we conducted a sensitivity analysis using nearest monitor data for PM_2.5_ and found similar, albeit attenuated, results (see Supplemental Material, Table S4). Furthermore, our models controlled for some of the same covariates that were in the air pollution prediction models, such as population density and land use, and still observed an association between air pollution and SES, indicating that SES is independently associated with a latent construct of air pollution.

Our study has implications for research on the health effects of air pollution. Differential exposures to air pollution, especially if extended over long periods, could have important implications for population health and health disparities. Although the associations we observed between NSES and air pollution were generally of small magnitude, they could confound associations of air pollution with health outcomes if the outcomes are associated with SES through mechanisms that do not involve air pollution as an intermediate. These confounding effects may be particularly important when the magnitude of associations of air pollution exposures with health outcomes is also expected to be small. Our results, therefore, suggest that studies of air pollution and health should consider adjusting for SES, especially NSES. However they also highlight the importance of investigating the SES–air pollution associations in specific settings because some heterogeneity of associations is to be expected. In fact, these results may not be generalizable to the U.S. population as a whole, or even to the larger metropolitan areas represented in the study, because participants were not evenly distributed throughout each MESA city.

SES is a powerful force that shapes exposure to a host of biomedical, environmental, and psychosocial factors that influence health (Link and Phelan 1995). Our results highlight how SES is associated with environmental exposures. In the specific case of air pollution exposures, those with higher SES can choose to live in homes further from the highway or leverage community resources to make improvements to air quality. We found that NSES was an especially important predictor of air pollution exposures. Investigating the drivers of these associations, while important, is beyond the scope of this study. A more comprehensive discussion of this issue can be found in Mohai et al. (2009). Research aimed at understanding the joint effects of air pollution and socioeconomic/psychosocial factors on health outcomes has the potential to integrate previously disparate fields of study (i.e., social and environmental epidemiology) (Clougherty and Kubzansky 2009; Morello-Frosch and Shenassa 2006), incorporate health into environmental justice research (Brulle and Pellow 2006), and produce a more comprehensive examination of environmental factors.

## Conclusions

We investigated the environmental burden of air pollution and found that overall, as NSES decreased, concentrations of air pollutants increased. Furthermore, the air pollution–SES association differed across a given metropolitan area, specifically for New York City, where we observed a positive association between pollutant concentrations and SES. Our results have implications for the confounding effects of SES in studies of air pollution and health and for understanding the possible contributions of air pollution to health disparities.

## Supplemental Material

(434 KB) PDF
