# Supplementary material for: Air Pollution and Individual and Neighborhood Socioeconomic Status: Evidence from the Multi-Ethnic Study of Atherosclerosis (MESA)
Source: Environ Health Perspect. 2013 Sep 27;121(11-12):1325–33. doi: 10.1289/ehp.1206337 (PMC3855503; doi:10.1289/ehp.1206337)
Supplement: (434 KB) PDF [file ehp.1206337.s001.508.pdf]

Environmental Health Perspectives doi:10.1289/ehp.1206337

Supplemental Material

Air Pollution and Individual and Neighborhood Socioeconomic Status: Evidence from the Multi-Ethnic Study of Atherosclerosis (MESA)

Anjum Hajat, Ana V. Diez-Roux, Sara D. Adar, Amy H. Auchincloss, Gina S. Lovasi, Marie S. O'Neill, Lianne Sheppard, and Joel D. Kaufman

Table of Contents

Supplemental Material, Methods 2
Socioeconomic status 2
Modeling approaches 3
Supplemental Material, Table S1: Differences from mean $PM_{2.5}$ and percent difference from geometric mean of NO_x associated with an increase in individual and neighborhood socioeconomic status (SES) characteristics estimated from crude and minimally adjusted intrinsic conditional autoregressive (ICAR) models 5
Supplemental Material, Table S2: Differences from mean $PM_{2.5}$ and percent difference from geometric mean of NO_x associated with an increase in individual and neighborhood socioeconomic status (SES) characteristics estimated from multi-level models 6
Supplemental Material, Table S3: Differences from mean $PM_{2.5}$ (μg/m^3) and NO_x concentrations (ppb) associated with an increase in individual and neighborhood socioeconomic status (SES) characteristic by metropolitan area 8
Supplemental Material, Table S4: Differences from mean $PM_{2.5}$ associated with an increase in individual and neighborhood socioeconomic status (SES) characteristics estimated from intrinsic conditional autoregressive (ICAR) models that use nearest AQS monitor data to represent $PM_{2.5}$ 10
References 11

Supplemental Material, Methods

In this supplemental methods section we provide additional detail on several SES measures and modeling approaches used in this study.

Socioeconomic status

Individual SES variables: In addition to the four individual SES variables described in the methods section (income, wealth, education and occupation), we also examined the income-wealth index and working outside the home. The income-wealth index was specified as a 9 point scale (0 being the lowest level of income and no assets and 8 being the highest level of income and all 4 assets) was the sum of a 5 category income variable and the 5 point wealth index. The income-wealth index was described in depth previously (Hajat et al. 2010). Participants who reported being employed either full or part-time or who were retired but working for pay or volunteering were classified as working outside the home, while those who never worked outside the home, were employed but not currently working, were unemployed or retired and not working were classified as not working outside the home. Work outside the home and the income-wealth index are not presented here because the other individual SES variables were more representative of the 4 domains of SES.

Neighborhood SES index: The 16 variables included in the principal components analysis were: percent of occupied housing units, percent of housing units that are owner occupied out of total housing units, median value of occupied housing units, percent of housing units without telephone, percent of housing units without vehicle, percent living in same house in 1995, percent of person 25 or older with at least high school education, percent of persons 25 or older with at least a Bachelor’s degree, percent unemployed among civilians 16 and over in the labor

force, percent of civilians 16 and over not in the labor force, percent with management, professional and related occupation, median household income, percent households with household income greater than $50,000, percent of households with interest, dividends, or net rental income, percent of households with public assistance and percent of persons below the poverty level.

Modeling approaches

Priors for ICAR model: Prior specifications for the conditional and marginal variance were set to 0.05. Prior specification can be difficult for the conditional variance because of its conditional rather than marginal interpretation (Fong et al. 2010). Thus a range of other priors were tested; they are: 0.5, 0.1, 0.01, 0.005 and 0.001. These priors were used for both the conditional and marginal variances. In general parameter estimates changed only at the third decimal place and beyond. These small changes would not affect the conclusions of the study.

Multilevel Models: In addition to the spatial ICAR models, we used multilevel models to account for the clustering of individuals within neighborhoods. Multilevel models assume that people living within the same census tracts are more similar to each other than persons living in different tracts, but do not make assumptions about the relationship between census tracts as ICAR models do. Multilevel models are similar to ICAR models in that they are also random effects models. αεβThe equation for the multilevel model is below:

$$Y_{ij} = [\gamma_{00} + \gamma_{01} NSES + \gamma_{02} area\ covariates + \beta_1 individual\ SES + \beta_2 individual\ covariates + a_{ij} + \varepsilon_i]$$

where

Y_{ij} = air pollution concentration for i^{th} person within census tract j

γ_{00} = average of the neighborhood level intercepts

γ_{01} = neighborhood SES characteristic for census tract j

γ_{02} = area level covariates for subject i within census tract j (i.e. population density, high density land use)

β_1 = coefficient for individual SES characteristic

β_2 = coefficients for individual level covariates (i.e. age, race/ethnicity and sex)

a_{ij} = within census tract error term (random effect)

ε_i = between census tract error term

Supplemental Material, Table S1: Differences from mean $PM_{2.5}$ and percent difference from geometric mean of NO_x associated with an increase in individual and neighborhood socioeconomic status (SES) characteristics estimated from crude and minimally adjusted intrinsic conditional autoregressive (ICAR) models [a]

SES variable	SD	Model 1[b]	Model 2[c]
**Difference from mean $PM_{2.5}$ ($\mu g/m^3$) (95% CI)**			
Individual SES			
Family income [d]	3.5	-0.05 (-0.07, -0.03)	-0.05 (-0.07, -0.03)
Wealth index [d]	1.3	-0.05 (-0.08, -0.03)	-0.05 (-0.07, -0.03)
Education [d]	2.4	-0.04 (-0.06, -0.03)	-0.04 (-0.06, -0.02)
Management occupation [e]	NA	-0.07 (-0.10, -0.03)	-0.06 (-0.10, -0.03)
Neighborhood SES [f]			
Median value of owner occupied homes ($)	204,345	0.09 (0.03, 0.15)	0.02 (-0.04, 0.07)
% not in poverty	11.4	-0.28 (-0.37, -0.20)	-0.34 (-0.40, -0.27)
Median household income ($)	20,469	-0.24 (-0.34, -0.15)	-0.33 (-0.40, -0.26)
% ≥ high school degree	16.7	-0.83 (-0.94, -0.71)	-0.59 (-0.66, -0.51)
% management occupations	17.9	-0.50 (-0.61, -0.39)	-0.48 (-0.56, -0.40)
NSES index	6.3	-0.31 (-0.42, -0.21)	-0.38 (-0.46, -0.31)
**% difference from geometric mean NO_x (95% CI)**			
Individual SES			
Family income [d]	3.5	-1.23 (-1.61, -0.84)	-1.33 (-1.75, -0.92)
Wealth index [d]	1.3	-1.54 (-1.95, -1.12)	-1.42 (-1.85, -1.00)
Education [d]	2.4	-1.17 (-1.54, -0.80)	-1.14 (-1.53, -0.75)
Management occupation [e]	NA	-1.09 (-1.76, -0.41)	-1.04 (-1.72, -0.37)
Neighborhood SES [f]			
Median value of owner occupied homes ($)	204,345	0.03 (-1.17, 1.24)	-2.81 (-3.91, -1.72)
% not in poverty	11.4	-9.60 (-11.29, -7.93)	-9.08 (-10.29, -7.87)
Median household income ($)	20,469	-7.64 (-9.44, -5.86)	-10.32 (-11.57, -9.07)
% ≥ high school degree	16.7	-17.47 (-19.57, -15.39)	-12.55 (-13.93, -11.18)
% management occupations	17.9	-7.76 (-9.81, -5.74)	-10.20 (-11.68, -8.72)
NSES index	6.3	-8.10 (-10.05, -6.18)	-11.04 (-12.43, -9.67)

[a] SES variables are scaled so higher values indicate higher SES. [b] Model 1: Crude model. [c] Model 2: adjusted for age, race/ethnicity, sex and metropolitan area. [d] Parameter estimates for family income, wealth index and education refer to a one unit increase in the z-score for these variables, which were originally ordinal variables that were transformed into z-scores (see methods section for more details). [e] Management occupation is dichotomous (management versus non-management occupations. [f] Parameter estimates for neighborhood SES variables refer to a one-standard deviation unit increase in that variable.

Supplemental Material, Table S2: Differences from mean $PM_{2.5}$ and percent difference from geometric mean of NO_x associated with an increase in individual and neighborhood socioeconomic status (SES) [a] characteristics estimated from multi-level models [b]

SES variable	Model 1[c]	Model 2[d]	Model 3[e]	Model 4[f]
**Difference from mean $PM_{2.5}$ ($\mu g/m^3$) (95% CI)**				
Individual SES				
Family income [g]	-0.05 (-0.07, -0.03)	-0.06 (-0.08, -0.04)	-0.05 (-0.07, -0.03)	-0.03 (-0.05, -0.01)
Wealth index [g]	-0.06 (-0.08, -0.04)	-0.06 (-0.08, -0.04)	-0.05 (-0.07, -0.03)	-0.03 (-0.05, -0.01)
Education [g]	-0.05 (-0.07, -0.04)	-0.05 (-0.07, -0.03)	-0.04 (-0.06, -0.02)	-0.03 (-0.05, -0.01)
Management occupation [h]	-0.07 (-0.11, -0.04)	-0.08 (-0.11, -0.04)	-0.06 (-0.1, -0.03)	-0.05 (-0.09, -0.02)
Neighborhood SES [i]				
Median value of owner occupied homes ($)	0.08 (0.02, 0.15)	0.02 (-0.04, 0.07)	0.02 (-0.04, 0.08)	0.003 (-0.05, 0.06)
% not in poverty	-0.34 (-0.42, -0.26)	-0.35 (-0.42, -0.29)	-0.34 (-0.41, -0.28)	-0.24 (-0.3, -0.17)
Median household income ($)	-0.27 (-0.36, -0.18)	-0.32 (-0.38, -0.25)	-0.31 (-0.37, -0.24)	-0.22 (-0.29, -0.15)
% ≥ high school degree	-0.94 (-1.04, -0.84)	-0.59 (-0.66, -0.52)	-0.57 (-0.65, -0.5)	-0.45 (-0.52, -0.38)
% management occupations	-0.55 (-0.66, -0.45)	-0.43 (-0.51, -0.36)	-0.42 (-0.50, -0.34)	-0.31 (-0.39, -0.24)
NSES index	-0.38 (-0.47, -0.28)	-0.36 (-0.43, -0.29)	-0.35 (-0.42, -0.27)	-0.27 (-0.34, -0.2)
**% difference from geometric mean NO_x (95% CI)**				
Individual SES				
Family income [g]	-1.30 (-1.69, -0.92)	-1.49 (-1.86, -1.11)	-1.42 (-1.83, -1.01)	-0.99 (-1.40, -0.60)
Wealth index [g]	-1.66 (-2.07, -1.25)	-1.65 (-2.06, -1.25)	-1.48 (-1.91, -1.06)	-0.97 (-1.38, -0.57)
Education [g]	-1.21 (-1.58, -0.84)	-1.33 (-1.70, -0.96)	-1.14 (-1.53, -0.75)	-0.88 (-1.26, -0.50)
Management occupation [h]	-1.30 (-2.02, -0.59)	-1.47 (-2.19, -0.77)	-1.20 (-1.92, -0.49)	-0.93 (-1.63, -0.24)
Neighborhood SES [i]				
Median value of owner occupied homes ($)	-0.47 (-1.67, 0.72)	-2.85 (-3.93, -1.79)	-2.78 (-3.85, -1.73)	-3.03 (-4.01, -2.06)
% not in poverty	-12.76 (-14.39, -11.17)	-9.63 (-10.79, -8.47)	-9.37 (-10.54, -8.22)	-6.81 (-7.86, -5.77)
Median household income ($)	-9.62 (-11.37, -7.90)	-10.41 (-11.58, -9.25)	-10.15 (-11.33, -8.99)	-7.65 (-8.69, -6.61)
% ≥ high school degree	-20.96 (-22.91, -19.03)	-13.02 (-14.32, -11.73)	-12.68 (-13.99, -11.38)	-9.52 (-10.68, -8.37)
% management occupations	-9.83 (-11.85, -7.85)	-9.71 (-11.10, -8.34)	-9.36 (-10.75, -7.98)	-6.92 (-8.13, -5.72)
NSES index	-10.56 (-12.48, -8.69)	-10.77 (-12.08, -9.49)	-10.45 (-11.75, -9.16)	-8.17 (-9.31, -7.05)

[a] All SES variables are scaled so higher values indicated higher SES. [b] Additional details on the multilevel model can be found in the Supplemental Material, methods section. [c] Model 1: Crude model. [d] Model 2: adjusted for metropolitan area. [e] Model 3: adjusted for age, race/ethnicity, sex and

metropolitan area. [f] Model 4: adjusted for variables in model 3 plus population density and high density land use. [g] Parameter estimates for family income, wealth index and education refer to a one unit increase in the z-score for these variables, which were originally ordinal variables that were transformed into z-scores (see methods section for more details). [h] Management occupation is a dichotomous variable (management versus non-management occupations). [i] Parameter estimates for neighborhood SES variables refer to a one-standard deviation unit increase in that variable.

Supplemental Material, Table S3: Differences from mean $PM_{2.5}$ ($\mu g/m^3$) and NO_x concentrations (ppb) associated with an increase in individual and neighborhood socioeconomic status (SES) characteristic by metropolitan area [a]

	Mean $PM_{2.5}$ (95% CI)		Mean NO_x (95% CI)	
**SES variable**	**Model 1[b]**	**Model 2[c]**	**Model 1[b]**	**Model 2[c]**
**Forsyth County, North Carolina**				
Family income [d]	-0.02 (-0.03, -0.01)	-0.02 (-0.03, -0.01)	-0.88 (-1.27, -0.48)	-0.86 (-1.29, -0.44)
Wealth index [d]	-0.02 (-0.03, -0.01)	-0.02 (-0.03, -0.002)	-0.99 (-1.42, -0.56)	-0.92 (-1.35, -0.48)
Median home value ($)	-0.05 (-0.22, 0.13)	-0.02 (-0.19, 0.16)	-5.35 (-10.34, -0.26)	-4.82 (-9.82, 0.29)
Median household income ($)	-0.05 (-0.10, 0.002)	-0.05 (-0.10, 0.01)	-2.39 (-3.83, -0.92)	-2.32 (-3.74, -0.87)
% ≥ high school degree	-0.09 (-0.16, -0.01)	-0.08 (-0.16, -0.004)	-2.98 (-5.06, -0.85)	-2.90 (-4.95, -0.81)
NSES index	-0.04 (-0.10, 0.02)	-0.03 (-0.09, 0.03)	-2.24 (-3.91, -0.52)	-2.12 (-3.79, -0.41)
**St. Paul, Minnesota**				
Family income [d]	-0.03 (-0.05, -0.02)	-0.03 (-0.04, -0.01)	-0.38 (-0.62, -0.14)	-0.35 (-0.62, -0.09)
Wealth index [d]	-0.06 (-0.08, -0.04)	-0.06 (-0.07, -0.04)	-0.75 (-1.02, -0.49)	-0.73 (-1.02, -0.44)
Median home value ($)	-0.56 (-0.83, -0.28)	-0.54 (-0.82, -0.26)	-11.76 (-16.07, -7.47)	-11.66 (-15.97, -7.37)
Median household income ($)	-0.33 (-0.39, -0.26)	-0.32 (-0.38, -0.26)	-4.70 (-5.60, -3.81)	-4.55 (-5.52, -3.61)
% ≥ high school degree	-0.47 (-0.56, -0.38)	-0.47 (-0.56, -0.38)	-5.67 (-7.12, -4.21)	-5.65 (-7.10, -4.19)
NSES index	-0.46 (-0.55, -0.37)	-0.45 (-0.54, -0.37)	-5.74 (-7.12, -4.37)	-5.66 (-7.05, -4.28)
**Baltimore, Maryland**				
Family income [d]	-0.03 (-0.07, -0.001)	-0.03 (-0.07, 0.005)	-0.11 (-0.47, 0.26)	-0.12 (-0.51, 0.28)
Wealth index [d]	-0.004 (-0.04, 0.03)	-0.001 (-0.04, 0.04)	-0.23 (-0.64, 0.17)	-0.23 (-0.63, 0.18)
Median home value ($)	-1.48 (-1.87, -1.08)	-1.47 (-1.87, -1.07)	-12.91 (-18.14, -7.58)	-12.83 (-18.07, -7.50)
Median household income ($)	-0.41 (-0.52, -0.30)	-0.40 (-0.51, -0.29)	-3.60 (-5.04, -2.13)	-3.56 (-5.01, -2.09)
% ≥ high school degree	-0.57 (-0.72, -0.41)	-0.56 (-0.72, -0.40)	-6.60 (-8.72, -4.43)	-6.60 (-8.74, -4.42)
NSES index	-0.63 (-0.79, -0.48)	-0.63 (-0.78, -0.48)	-6.11 (-8.11, -4.08)	-6.10 (-8.12, -4.06)
**Chicago, Illinois**				
Family income [d]	-0.01 (-0.03, 0.01)	-0.01 (-0.04, 0.01)	0.05 (-0.18, 0.29)	0.06 (-0.19, 0.31)
Wealth index [d]	-0.001 (-0.02, 0.02)	-0.001 (-0.02, 0.02)	0.40 (0.15, 0.65)	0.42 (0.16, 0.67)
Median home value ($)	-0.07 (-0.14, -0.01)	-0.07 (-0.13, -0.01)	-1.11 (-1.78, -0.43)	-1.04 (-1.71, -0.37)
Median household income ($)	-0.32 (-0.40, -0.23)	-0.321 (-0.40, -0.23)	-3.06 (-3.84, -2.26)	-2.89 (-3.68, -2.08)
% ≥ high school degree	-0.47 (-0.61, -0.33)	-0.48 (-0.61, -0.34)	-5.60 (-6.80, -4.35)	-5.73 (-6.90, -4.51)
NSES index	-0.30 (-0.40, -0.20)	-0.30 (-0.40, -0.20)	-3.62 (-4.55, -2.67)	-3.49 (-4.42, -2.55)
**Los Angeles, California**				
Family income [d]	-0.04 (-0.08, 0.01)	-0.04 (-0.10, 0.01)	-1.02 (-1.51, -0.54)	-1.11 (-1.66, -0.57)

SES variable	Mean $PM_{2.5}$ (95% CI) Model 1[b]	Model 2[c]	Mean NO_x (95% CI) Model 1[b]	Model 2[c]
Wealth index [d]	-0.02 (-0.08, 0.03)	-0.03 (-0.08, 0.02)	-1.26 (-1.77, -0.76)	-1.28 (-1.83, -0.73)
Median home value ($)	0.33 (0.03, 0.63)	0.30 (0.003, 0.60)	-7.41 (-10.2, -4.63)	-6.10 (-8.88, -3.35)
Median household income ($)	-0.11 (-0.25, 0.03)	-0.16 (-0.30, -0.01)	-7.80 (-9.01, -6.57)	-7.23 (-8.47, -5.99)
% ≥ high school degree	-0.34 (-0.48, -0.21)	-0.35 (-0.48, -0.22)	-7.20 (-8.35, -6.05)	-6.98 (-8.11, -5.85)
NSES index	-0.20 (-0.38, -0.03)	-0.24 (-0.41, -0.06)	-9.18 (-10.62, -7.73)	-8.60 (-10.06, -7.13)
**New York, New York**				
Family income [d]	-0.18 (-0.28, -0.08)	-0.13 (-0.24, -0.01)	-0.74 (-1.49, 0.02)	-0.48 (-1.35, 0.39)
Wealth index [d]	-0.23 (-0.33, -0.13)	-0.20 (-0.30, -0.09)	-0.86 (-1.62, -0.09)	-0.72 (-1.52, 0.08)
Median home value ($)	0.16 (0.04, 0.28)	0.16 (0.04, 0.28)	0.16 (-0.66, 0.97)	0.18 (-0.63, 0.99)
Median household income ($)	0.32 (0.07, 0.57)	0.35 (0.10, 0.60)	3.38 (1.76, 5.05)	3.42 (1.79, 5.09)
% ≥ high school degree	0.10 (-0.20, 0.39)	0.14 (-0.16, 0.43)	0.98 (-0.89, 2.85)	1.05 (-0.84, 2.97)
NSES index	0.42 (0.17, 0.66)	0.43 (0.19, 0.68)	2.49 (0.93, 4.05)	2.54 (0.97, 4.12)

[a] SES variables are scaled so higher values indicated higher SES. [b] Model 1: crude model. [c] Model 2: adjusted for age, race/ethnicity and sex. [d] Parameter estimates for family income and wealth index refer to a one unit increase in the z-score for these variables, which were originally ordinal variables that were transformed into z-scores (see methods section for more details).

Supplemental Material, Table S4: Differences from mean $PM_{2.5}$ associated with an increase in individual and neighborhood socioeconomic status (SES) characteristics [a] estimated from intrinsic conditional autoregressive (ICAR) models that use nearest AQS monitor data to represent $PM_{2.5}$ [b]

SES variable	Model 1[c]	Model 2[d]	Model 3[e]	Model 4[f]
**Difference from mean $PM_{2.5}$ ($\mu g/m^3$) (95% CI)**				
Individual SES				
Family income [g]	-0.009 (-0.02, -0.002)	-0.009 (-0.02, -0.002)	-0.007 (-0.01, 0.0005)	-0.006 (-0.01, 0.002)
Wealth index [g]	-0.003 (-0.01, 0.005)	-0.004 (-0.01, 0.004)	-0.001 (-0.009, 0.007)	0.0004 (-0.007, 0.008)
Education [g]	-0.007 (-0.01, -0.0007)	-0.009 (-0.02, -0.002)	-0.006 (-0.01, 0.001)	-0.005 (-0.01, 0.002)
Management occupation [h]	-0.005 (-0.02, 0.007)	-0.003 (-0.02, 0.009)	-0.005 (-0.02, 0.007)	-0.006 (-0.02, 0.006)
Neighborhood SES [i]				
Median value of owner occupied homes ($)	-0.02 (-0.04, 0.005)	-0.03 (-0.06, -0.01)	-0.03 (-0.06, -0.01)	-0.03 (-0.06, -0.01)
% not in poverty	-0.01 (-0.05, 0.02)	-0.08 (-0.10, -0.05)	-0.07 (-0.10, -0.04)	-0.07 (-0.10, -0.04)
Median household income ($)	0.004 (-0.03, 0.04)	-0.08 (-0.12, -0.05)	-0.08 (-0.11, -0.05)	-0.08 (-0.11, -0.04)
% ≥ high school degree	-0.08 (-0.13, -0.04)	-0.12 (-0.16, -0.08)	-0.12 (-0.16, -0.08)	-0.12 (-0.16, -0.08)
% management occupations	-0.03 (-0.08, -0.008)	-0.07 (-0.11, -0.03)	-0.07 (-0.11, -0.03)	-0.06 (-0.10, -0.03)
NSES index	-0.04 (-0.08, 0.003)	-0.10 (-0.13, -0.06)	-0.10 (-0.13, -0.06)	-0.09 (-0.13, -0.06)

[a] All SES variables are scaled so higher values indicated higher SES. [b] Pollutant concentrations for the year 2000 estimated from nearest Environmental Protection Agency's Air Quality System (AQS) monitor to participants home address. [c] Crude model [d] Metropolitan area adjusted model [e] Model adjusted for age, race/ethnicity, sex and metropolitan area [f] Model adjusted for variables in model 3 plus population density and high density land use. [g] Parameter estimates for family income, wealth index and education refer to a one unit increase in the z-score for these variables, which were originally ordinal variables that were transformed into z-scores (see methods section for more details). [h] Management occupation is a dichotomous variable (management versus non-management occupations). [i] Parameter estimates for neighborhood SES variables refer to a one-standard deviation unit increase in that variable.

References

Fong Y, Rue H, Wakefield J. 2010. Bayesian inference for generalized linear mixed models. Biostatistics 11: 397-412.

Hajat A, Diez-Roux A, Franklin TG, Seeman T, Shrager S, Ranjit N, et al. 2010. Socioeconomic and race/ethnic differences in daily salivary cortisol profiles: The Multi-Ethnic Study of Atherosclerosis. Psychoneuroendocrinology 35: 932-943.
